# A preliminary study on the feasibility of community game-based respiratory muscle training for individuals with high cervical spinal cord injury levels: a novel approach

**DOI:** 10.1186/s13102-022-00534-x

**Published:** 2022-07-22

**Authors:** Dongheon Kang, Jiyoung Park, Seon-Deok Eun

**Affiliations:** Department of Healthcare and Public Health Research, National Rehabilitation Hospital, Ministry of Health and Welfare, Seoul, 01022 South Korea

**Keywords:** Spinal cord injury, Community program, Respiration, Respiratory function tests, Respiratory muscle training

## Abstract

**Background:**

Respiratory disorders result in rehospitalization and premature death of patients with cervical spinal cord injuries (CSCI). Community game-based respiratory muscle training (RMT) programs could reduce secondary complications.

**Methods:**

We examined the feasibility and preliminary efficacy of RMT as a community-based exercise program. Among the 10 included participants (eight male and two female), four, one, one, and four reported C3, C4, C5, and C6 complete injuries, respectively (eight graded by American Spinal Injury Association impairment scale [ASIA] A and two by ASIA B). Their mean age was 43 ± 12.3 y. The time since injury was 10 ± 6.7 y. The participants completed an RMT program for 60 min/day, twice weekly, for 8 weeks. The participants were trained in the use of a newly developed game-based RMT device. The device provides consistent pressure for respiratory muscle strength and endurance training. Seven RMT devices were modified to allow 10 game-based RMT programs. Forced vital capacity (FVC), forced expiratory volume in 1 s (FEV_1_), peak expiratory flow (PEF), vital capacity (VC), inspiratory capacity (IC), inspiratory reserve volume (IRV), expiratory reserve volume (ERV), maximum inspiratory pressure (MIP), maximum expiratory pressure (MEP), and peak cough flow (PCF) were measured.

**Results:**

There were improvements after RMT compared to pre-RMT in FVC (*p* = 0.027, 10.62%, 0.22 effect size [ES]), PEF (*p* = 0.006, 23.21%, 0.45 ES), VC (*p* = 0.002, 35.52%, 0.60 ES), IC (*p* = 0.001, 46.94%, 0.81 ES), IRV (*p* = 0.001, 90.53%, 1.22 ES), MIP (*p* = 0.002, 97.25%, 1.32 ES), MEP (*p* = 0.005, 141.12%, 1.07 ES), and PCF (*p* = 0.001, 35.60%, 0.74 ES). The participants reported a positive impact of the program.

**Conclusions:**

Community game-based RMT for individuals with CSCI appears to be safe and feasible. Community exercise with RMT use may have a positive impact on the respiratory measures for patients with CSCI who are vulnerable to respiratory compromise.

***Trial registration*:**

KCT0005980.

## Background

Spinal cord injury (SCI) has become increasingly widespread [1]. Falls, violence, and motor vehicle accidents are the most common causes of SCI [2]. With advances in medical technology and care over the past decades, the associated increase in life expectancy has led to a rising population of patients with SCI [3]. Further, these patients may have an increased risk of acquiring potentially fatal secondary health conditions [4, 5].

Dysfunctions owing to cervical SCI (CSCI) include complete or partial impairment of motor control and sensory function [6]. CSCI disrupts the respiratory function of inspiratory and expiratory muscles such as the diaphragm, intercostal muscles, accessory respiratory muscles, and abdominal muscles [7, 8]. The reduction of respiratory function is considered the major short- and long-term cause of morbidity and mortality in SCI cases owing to the associated complications, such as atelectasis or pneumonia [9, 10].

A common consequence of CSCI is the defective innervation of the inspiratory and expiratory muscles [11]. This defective innervation results in muscle dysfunction that contributes to changes in the chest wall compliance, lung capacity, ventilatory efficiency, and maximum expiratory and inspiratory muscle pressure [11]. Particularly, in CSCI cases, there is impairment in the control of the cervical spinal cord over the respiratory muscles located below the injury point [11]. The resulting paralysis of the respiratory muscles reduces the ability to cough and accumulates airway secretions, thus causing various respiratory complications [12]. Furthermore, weakened respiratory muscles cannot sufficiently inflate the lung to its maximum volume; in addition, they cannot compress the lung to its minimum residual volume [13]. Therefore, prolonged insufficient thoracic expansion results in the shortening and hardening of the thoracic tissue and in muscular fibrosis, which reduces the compliance of the thoracic cavity and promotes atelectasis; in turn, it results in a lower compliance of the lungs [13, 14]. Such factors may reduce coughing and sputum secretion abilities, which may pose as serious disturbances in respiratory hygiene [15].

In the initial stage of spinal shock, pulmonary function is reduced due to flaccid paralysis of all the muscles and paralysis of the respiratory muscles [16]. In patients with CSCI, recovery to the pre-SCI state of respiratory function is difficult. Individuals with CSCI may experience a vital capacity reduction of up to 50% and a functional residual capacity reduction of up to 75% [8]. As the reduced respiratory function can restrict the daily life of individuals with CSCI with challenges (i.e., dyspnea) and difficulties in sputum secretion [17], respiratory muscle training (RMT) seems to be essential to boost impaired pulmonary function and reduce respiratory complications [18, 19]. RMT demonstrated significant improvements in respiratory muscle strength and endurance, thereby ameliorating respiratory complications [16, 17].

Although the implementation of RMT interventions is crucial to prevent respiratory complications following CSCI, participation in irregular RMT intervention may lead to obstructive pulmonary disease and worsening of respiratory failure [20]. In particular, as respiratory failure in individuals with CSCI increases the risk of respiratory complications, early implementation of the appropriate RMT intervention seems to be essential [16]. RMT interventions result in the improvement of respiratory function, effective coughing for the removal of secretions, and reduced secretions owing to autonomic dysfunction [15].

According to a Cochrane review [16], several studies have explored the mechanism of respiratory dysfunction and conducted various RMT interventions to improve respiratory function [16]; 11 studies have demonstrated that such interventions were safe and effective in improving the respiratory strength and coughing ability of patients with CSCI. Nevertheless, interventions to improve respiratory strength and coughing ability in patients with CSCI were usually performed in a hospital setting by performing everyday activities, such as blowing candles, blowing balloons, blowing a ping-pong ball, and singing, without using specialized medical devices [16, 21]. Although Berlowitz and Tamplin advise repetitive RMT interventions [16], the procedure is considered monotonous and the level of improvement cannot be assessed during the intervention. Breathing training is thus inconvenient. Game-based RMT was developed to overcome these issues and provide engaging and more practical breathing training; this program incorporated the term “game” into RMT [22]. It was created to allow individuals with CSCI to be excited and engaged, thus enabling the easier performance of RMT within the community. This program enables the continuous management of individuals with CSCI to prevent respiratory complications, along with the maximization of RMT. Our aim was to examine the feasibility and preliminary efficacy of game-based RMT on respiratory function and cough ability in individuals with CSCI.

## Methods

### Participants

**Fig. 1:**
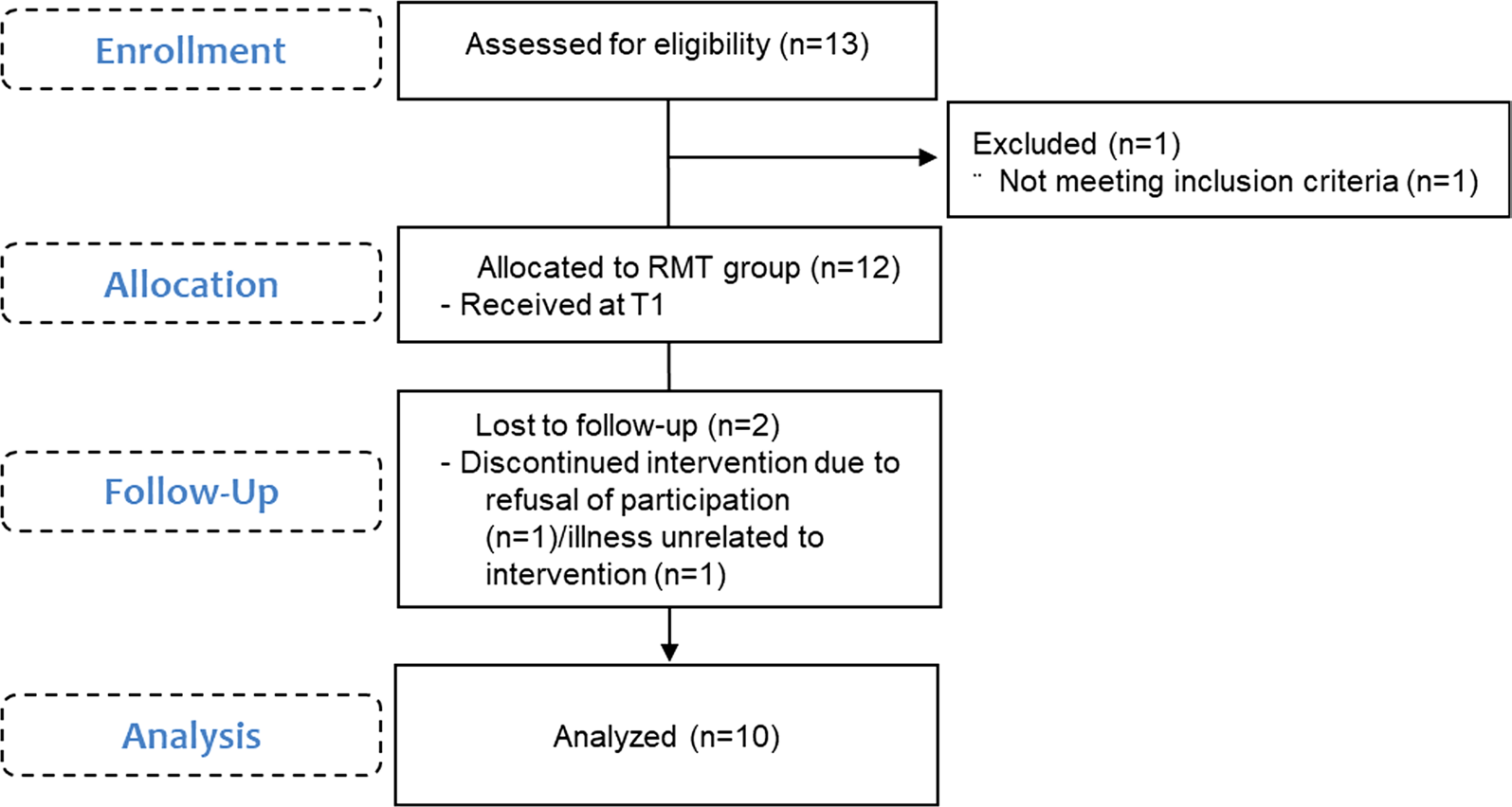
Consolidated Standards of Reporting Trials (CONSORT) diagram showing the flow of subjects through each stage of the study

This study was approved, and all methods were carried out in accordance with relevant guidelines and regulations by the National Rehabilitation Hospital’s Institutional Review Board (NRCIRB 2016-03-029). In this feasibility and preliminary study, participants were recruited on a voluntary basis from a rehabilitation sport (RS) class. This study was conducted following the principles of the Declaration of Helsinki. The study protocol was registered and assigned the number KCT0005980 (first registration 09/03/2021). Both verbal and written consent for study participation was obtained from each participant prior to the commencement of the study. All the participants were informed of the objectives, procedures, and potential risks or discomfort associated with study participation. The RS class was completed at The Korea National Rehabilitation Institute Project (Seoul, South Korea), a community-based organization that provides exercise opportunities for people with disabilities. The RS class was specifically designed for patients with CSCI and included all such cases irrespective of their age, time since injury, and injury levels. All the participants in the RS class were allowed to participate in this study (Fig. 1); volunteers for the study signed an informed consent. The participants were included in the study if they had a CSCI and an American Spinal Injury Association (ASIA) impairment scale level of A or B. Additionally, the participants were included in the study if they were over 20 years of age. We excluded those who had any other neurological condition other than SCI; could not complete a single repetition using the RMT device; had no arthritis or neuromuscular disease in the spine that could affect lung function; or had any conditions limiting participation in exercise, including but not limited to orthopedic, cardiac, or pulmonary diseases. Consenting participants self-reported the level of injury, complete versus incomplete, ASIA level, age, and time since injury.

### Interventions

#### Rehabilitation sport class

The RS class was held at the Korea National Rehabilitation Institute Project twice per week for 8 consecutive weeks. The program consisted of a warm-up, cool-down (accessory muscles stretching), game-based RMT program, and arrangement exercise (breathing assistant muscle stretching).

#### Game-based respiratory muscle training

The participants were trained in the use of a developed game-based RMT device [22]. The game-based RMT device provides consistent pressure for respiratory muscle strength and endurance training, regardless of the breathing speed. The programs comprised 8 game-based RMT interventions for both inspiratory and expiratory muscle training. The program was conducted using 3 or 4 devices per training day. The details of the programs performed in this study are presented in Table 1.

**Table 1:** Game-based respiratory muscle training

Contents		Explanation	Expected effect
Breath goal-in		An exercise where the participant needs to blow on a mini-ball and score a goal by avoiding moving obstacles. The game can be played alone or with up to four players	Inspiration (volume, strength, and peak) and expiration (volume and peak)
Breathing billiard		A mini-pool table was created by reducing the area of original-sized pool tables. The aim of this exercise was to push balls of different weights by breathing. This game can be performed alone or with up to four people	Inspiration (volume), expiration (peak), and neck movement
Multi-play ball	Lift ball competition	The goal of this exercise was to lift the ball by breathing into a cylinder. After the start signal, the distance from the bottom of the cylinder to the bottom part of the ball was measured using a ruler attached to the acrylic cylinder body	Inspiration (volume) and expiration (volume, strength, and peak)
	Ball reciprocating count battle	This exercise measured the number of times that the sound sensor was touched by the ball for 7 s using a cylinder with a sound sensor attached	Inspiration (volume and strength) and expiration (volume, strength, and peak)
	Holding the ball	This exercise measured the number of times that the sound sensor was touched by the ball for 7 s using a cylinder with a sound sensor attached	Inspiration (volume) and expiration (volume and strength)
Breathing tug-of-war		A two-player exercise where players need to breathe and move the ball toward their side for 3 s	Inspiration (volume, strength, and peak) and expiration (volume, strength, and peak)
Breathing marble		A board-game-like breathing exercise where missions need to be completed	Inspiration (volume) and expiration (volume and peak)
Breathing curling		A modified version of curling where the stone is blown toward the target board	Inspiration (volume) and expiration (volume, strength, and peak)
Boroling		A game that combines curling and bocci and is a sport for those with severe disabilities	Inspiration (volume), expiration (volume and strength), and neck movement
Blowgun dart		A game where blowgun bullets with suction plates are blown using the blowgun to hit a target with a suction plate	Inspiration (volume), expiration (peak), and neck movement

An exercise instructor and physical therapist were trained in the administration of the RMT devices; the initial training level was set such that they could complete 15 breaths/set without exhibiting symptoms of hyperventilation. The RMT program (60 min/session) was performed twice a week (8 weeks) using the developed RMT devices. Each training day program consisted of warm-up (10 min) with stretching muscles around the neck, cool-down (10 min), and game-based RMT (40 min).

This training protocol was reported to be feasible and effective in patients with CSCI [22]. The participants were provided weekly training diaries noting the training repetition and set number, perceived rate of exertion, and adverse responses. If a training diary was forgotten, the report was recorded by an exercise instructor. All the participants received their own exercise instructor, and the resistance was advanced weekly.

### Measures

The outcome measures were evaluated before and after the 8-week program. Before the evaluation, the participants were taught regarding the test method. The respiratory function was measured using a digital respiratory function measuring device (Pony FX, COSMED, Rome, Italy) [22]. For accurate measurement, the examiner provided sufficient explanation to the participant to comprehend the test, presented the process, and measured the respiratory function [23]. In this study, the respiratory function of forced vital capacity (FVC), forced expiratory volume in 1 s (FEV_1_), peak expiratory flow (PEF), vital capacity (VC), inspiratory capacity (IC), inspiratory reserve volume (IRV), and expiratory reserve volume (ERV) were measured.

The respiratory muscle strength was evaluated by the maximum inspiratory pressure (MIP) and the maximum expiratory pressure (MEP) measurement using the Pony FX (COSMED) [16].

The peak cough flow (PCF), which measures the ability to produce an effective cough, was assessed using a PCF meter (PF100, Microlife Corp., Cambridge, UK). The participants were instructed to inhale to the maximum capacity and then cough as strongly as possible [16]. Three measurements of each variable were obtained and the mean of the three values was analyzed.

### Data analysis

All the statistical analyses were performed with SPSS version 21.0 (IBM Corp., Armonk, NY). The means and standard deviations of each variable were obtained using descriptive statistics. The number of samples (n = 10) was < 30. The normality test was performed using the Shapiro–Wilk test; however, the non-parametric test was conducted, since it satisfied the significance level (p < 0.05). The Wilcoxon test was conducted to assess the differences between the pre- and post-exercise measurements of the participants’ performance. In addition to this null hypothesis testing, the data were also assessed for clinical significance using an approach based on the magnitudes of change. We calculated the magnitude of the size of differences by the effect size (ES) [24]. We considered an ES of 0.00–0.19, 0.20–0.49, 0.50–0.79, and ≥ 0.80 as trivial, small, moderate, and high, respectively [24].

## Results

### Demographics

In total, 10 participants (sex, eight male and two female) with CSCI consented to participate, completed the training, and were included in the analysis. All participants had complete cervical level injuries (Table 2). Their age was 43.6 ± 12.3 years (mean ± standard deviation), and the time since injury onset was 10.3 ± 6.7 years (Table 2). Pre- and post-measures were obtained for all participants who completed the training.

**Table 2:** Participant demographics

Participant	Level	C/I	ASIA	Years post	Age	Sex
1	C3	C	B	4	32	M
2	C6	C	A	7	49	M
3	C3	C	A	13	35	M
4	C6	C	A	11	34	M
5	C6	C	B	6	28	M
6	C3	C	A	23	42	M
7	C3	C	A	2	61	M
8	C5	C	A	19	38	M
9	C4	C	A	7	57	F
10	C6	C	A	7	60	F

Level, injury level; C, complete injury; I, incomplete injury; ASIA, American Spinal Injury Association impairment scale level as reported by the participant; Years post, years post injury; F, female; M, male

### Outcome measures

The mean difference for all measures across participants demonstrated an overall improvement in all the outcome measures (Table 3). In the respiratory assessment, FVC (*p* = 0.027, 10.62%, 0.22 ES), PEF (*p* = 0.006, 23.21%, 0.45 ES), VC (*p* = 0.002, 35.52%, 0.60 ES), IC (*p* = 0.001, 46.94%, 0.81 ES), and IRV (*p* = 0.001, 90.53%, 1.22 ES) were significantly improved. MIP (*p* = 0.002, 97.25%, 1.32 ES) and MEP (*p* = 0.005, 141.12%, 1.07 ES) significantly improved after the game-based RMT. The PCF values (*p* = 0.001, 35.60%, 0.74 ES) also significantly improved.

**Table 3:** Summary of training effects for respiratory function test variables obtained before and after intervention

Variables	Before intervention, mean ± SD	After intervention, mean ± SD	% Change (95% CI)		T	*p*	Effect size (Cohen’s d)
FVC (L)	2.26 ± 1.09	2.50 ± 1.14*	10.62 (− 0.45, − 0.04)		− 2.65	0.027	0.22^#^
FEV_1_ (L)	1.96 ± 0.91	2.11 ± 0.92	7.65 (− 0.29, − 0.00)		− 2.25	0.051	0.16
PEF (L/sec)	4.48 ± 2.08	5.52 ± 2.54**	23.21 (− 1.69, − 0.38)		− 3.58	0.006	0.45^#^
VC (L)	1.83 ± 0.93	2.48 ± 1.21**	35.52 (− 0.99, − 0.31)		− 4.33	0.002	0.60^##^
IC (L)	1.47 ± 0.68	2.16 ± 0.98**	46.94 (− 0.99, − 0.37)		− 5.00	0.001	0.81^###^
IRV (L)	0.95 ± 0.47	1.81 ± 0.88**	90.53 (− 1.24, − 0.47)		− 4.98	0.001	1.22^###^
ERV (L)	0.39 ± 0.37	0.37 ± 0.40	− 5.13 (− 0.24, 0.27)		0.15	0.884	0.05
MIP (cmH_2_O)	29.10 ± 16.97	57.40 ± 25.03**	97.25 (− 42.82, − 13.78)		− 4.41	0.002	1.32^###^
MEP (cmH_2_O)	21.40 ± 21.87	51.60 ± 33.49**	141.12 (− 48.81, − 11.59)		− 3.67	0.005	1.07^###^
PCF (L/min)	194.10 ± 82.24	263.20 ± 104.40**	35.60 (− 103.10, − 35.10)		− 4.60	0.001	0.74^##^

Values are presented as means ± standard deviations. The *p* value was derived from a paired t-test of the results before and after the intervention

CI, confidence interval; FVC, forced vital capacity; FEV_1_, forced expiratory volume in one second; PEF, peak expiratory flow; VC, vital capacity; IC, inspiratory capacity; IRV, inspiratory reserve volume; ERV, expiratory reserve volume; MIP, maximum inspiratory pressure; MEP, maximum expiratory pressure; PCF, peak cough flow; MBorg, Modified Borg scale; SD, standard deviation

*p < 0.05; **p < 0.01

^#^Small, ^##^Moderate, ^###^Large

### Safety, feasibility, adverse events

There was no study related to adverse events. Respiratory variables were examined for the 10 participants who completed 16 training sessions during the 8 weeks (Table 3).

## Discussion

In this study, we reported that game-based RMT significantly improved the respiratory outcomes in patients with CSCI. This is the first study to demonstrate how a novel approach to game-based RMT with a community exercise program could be an encouraging intervention strategy for patients with CSCI.

A functional, effective method of RMT is required to support the repetitive, intensive training warranted for the respiratory rehabilitation of patients with CSCI. Additionally, participants’ active participation throughout the lengthy rehabilitation.

RMT should easily attract the interest of participants. Patients with CSCI experience weakened respiratory muscles, leading to ineffective cough or sputum removal capacity [16]. Furthermore, secretions accumulate in the airway owing to dysphagia and inspiration, leading to various respiratory complications, such as pneumonia and atelectasis [16]. Thus, RMT interventions based on an accurate diagnosis of functional capacity and condition, prognosis, and severity in patients with CSCI are vital [25, 26]. The effects of RMT on overall respiratory function were quantitatively examined by performing a comparison between the pre-exercise and post-exercise values. Interestingly, most outcome measures were improved, except for FEV_1_ and ERV.

The program was conducted in two 60-min sessions per week, for 8 weeks. To examine the effectiveness of RMT, pre- and post-intervention evaluations of the participants’ respiratory function, respiratory muscle strength, and cough ability were evaluated. An observation of the study findings revealed significant improvements in the FVC, PEF, VC, IC, IRV, MIP, MEP, and PCF throughout the 8-week RMT intervention period.

After training, both the FVC and FEV_1_ increased. The pulmonary function test (FVC and FEV_1_) is the simplest and most comprehensive respiratory functional assessment for the diagnosis and evaluation of airway diseases. The FVC and FEV_1_ measure forceful expiration following maximum inspiration and forceful expiration in one second, respectively [27]. Pulmonary function testing is used to measure the change and improvement of respiratory function in individuals with CSCI.

The observed significant improvement in FVC was consistent with the results of a previous study [19] that reported a similar improvement in the FVC of patients with SCI after 8 weeks of RMT training. However, this study used a combination of developed game-based RMT interventions instead of a traditional RMT. In addition, patients with CSCI (ASIA A or B) who have difficulty recruiting participants were included. The game-based RMT was used to strengthen the muscles involved in inhalation and exhalation, which resulted in an increase in the FVC and FEV_1_. The observed significant improvements suggested that game-based RMT interventions are effective in increasing the FVC in patients with CSCI.

After RMT completion, PEF, VC, IC, and IRV were significantly improved.

These results were similar to those of previous studies that reported a significant improvement in the PEF, VC, and IC after RMT in patients with SCI [10, 16, 28]. These results have been attributed to the changes in the chest wall characteristics [29], such as the recovery of diaphragmatic function [9, 30], improved ability of the accessory respiratory muscles, and increased stability and adaptability of the thoracic cavity [31]. Moreover, VC and expiratory flow decrease after CSCI, which can cause severe respiratory failure [32]; therefore, patients with CSCI should be managed with a steady RMT protocol to restore respiratory muscle function.

Evaluation of respiratory muscle strength is used to determine respiratory failure [33] and to evaluate the changes and improvement in coughing capacity in patients with SCI. Furthermore, the maximum cough flow is produced by an increase in the abdominal and chest pressure, generated by the contraction of the internal intercostal and abdominal muscles [34]. This method of evaluation is used as a measure to evaluate the degree and change in the improvement of the respiratory muscle strength and coughing capacity of patients with SCI [35]. Following 8 weeks of game-based RMT, improvements were observed in the MIP and MEP. The results of providing game-based RMT to patients with CSCI revealed significant improvements in the MIP and MEP. Our findings were consistent with those of a previous study [10], where significant improvements in the MIP and MEP were observed following 6 weeks of RMT among patients with spinal injuries (C4-C7, T1). These findings highlighted the possibility that the game-based RMT helped strengthen the muscles involved in inspiration and expiration, thus, improving the MIP and MEP. The improvements in the MIP and MEP may be attributed to the hyperventilation that occurred due to the participants’ efforts to win, a part of the RMT protocol, which might have helped activate and strengthen the respiratory muscles of the participants [22]. Thus, the RMT intervention program proposed in this study could be used to effectively improve the MIP and MEP.

Furthermore, the provision of game-based RMT for 8 weeks among patients with CSCI showed a significant improvement in the PCF. This finding was similar to that of a previous study that reported a significant improvement in the PCF following 4 weeks of RMT among patients with CSCI [36]. However, they used activation of the abdominal muscles using functional electrical stimulation with assisted RMT to improve the PCF. Coughing is an important protective mechanism to excrete secretions in an effort to prevent respiratory complications, such as atelectasis and pneumonia [37]. In order to cough effectively, the three stages of coughing (inhalation-compression-exhalation) should function normally [38]. Nevertheless, in cases where the spinal cord is damaged, the coughing mechanism could be abnormal, thus, requiring assistance in coughing to adequately excrete the secretions settling in the airway [15]. Intercostal and abdominal muscle paralysis makes expiratory muscle contraction following inhalation challenging [15]. Thus, the expiratory muscle contraction owing to lung expansion from the inspired air and chest wall recoil makes it more difficult for patients to cough effectively [39, 40]. Therefore, game-based RMT may be an effective intervention for the improvement of expiratory function and cough ability in patients with CSCI.

Conventional RMTs, such as diaphragmatic breathing, isocapnic hyperpnea training, air stacking exercise, pursed-lip breathing, and air-shifting, are repetitive hospital-based interventions, which could result in the loss of patients’ interest and in abandonment of the rehabilitation process [21]. The interventions suggested in a previous study [21] were mundane and did not demonstrate observable improvements, which resulted in challenges to the implementation of RMT. In this study, game-based voluntary hyperventilation was encouraged during RMT. The intervention was developed in an effort to alleviate the challenges and provide a more interesting and effective RMT procedure. Moreover, a combination of both game and RMT possesses several advantages compared to the conventional RMT protocols. First, game-based RMT can induce competition against other participants, thus promoting a more continuous participation in the program. Second, it provides an interactive environment for participants with the same condition. Third, the effectiveness of the RMT seems to be higher because of the increased interest and participation rate, which ultimately can improve the quality of life of patients with CSCI.

## Limitations

Since this was a preliminary pilot study, there were some limitations that should be acknowledged. First, it had a single group pre- and post-design, and there was no control group to compare the exercise effects. It is necessary to study the effects of RMT in future studies based on the principle of random allocation of participants with a control group. Second, the study had a small sample size and the participants were mostly male; thus, the results may not be generalizable to all patients with CSCI. Further studies should include more women to examine sex differences in the results of the respiratory function tests performed in patients with CSCI. Third, it is necessary to examine the effect of age, smoking status, location of injury (e.g., C4–C7), and onset of injury on the participants’ respiratory function. Fourth, the results did not indicate whether the improvements resulted from the performed exercise programs, as all the participants partook in various programs. Further studies with larger numbers of individuals with CSCI considering various injury characteristics (i.e., level, severity, and duration) and clinical information (i.e., smoking, tracheostomy, and use of ventilator) are warranted to identify the factors contributing to pulmonary function improvement and, ultimately, obtain favorable rehabilitative outcomes.

## Conclusions

To our knowledge, this is the first feasibility and preliminary study to suggest the use of RMT in combination with a community exercise program for patients with CSCI. Overall, the participants demonstrated improvement in all the respiratory outcomes. With increased education and expansion of these types of programs, compliance with an RMT program may increase.

Finally, we judged that the safety verification for the mechanical part should be carried out for the generalization of the developed RMT devices.

## Data Availability

The datasets used and/or analyzed during the current study are available from the corresponding author on reasonable request.

## Abbreviations

CSCI: Cervical spinal cord injury
ERV: Expiratory reserve volume
FVC: Forced vital capacity
FEV_1_: Forced expiratory volume in 1 s,
IC: Inspiratory capacity
IRV: Inspiratory reserve volume
MIP: Maximum inspiratory pressure
MEP: The maximum expiratory pressure
PEF: Peak expiratory flow
RMT: Respiratory muscle training
SCI: Spinal cord injury
VC: Vital capacity
